# The Buffalo Medical Library Association

**Published:** 1883-01

**Authors:** 


### The Buffalo Medical Library Association.

Under "Society Reports" will be found the proceedings for the organization of a Medical Library Association, a movement called forth by an urgent necessity long felt by the medical profession in this city. We are gratified to witness the enthusiasm with which the profession enters into the formation of this society, and to give our endorsement to the ultimate objects for which the society is called into existence. Heretofore the severe conservatism of our city in its commercial relations has extended its influence to all efforts upon the part of the few to provide facilities for the advancement of the interests of scientific and professional study. A sudden breaking loose from these hide-bound ideas has given our city a great impetus in its financial and commercial interests and progress. A like zeal is breaking out in professional circles, and among the evidences of this new life in the profession is the demand for greater library facilities than the private libraries afford, for reference, study and research. The interest is so wide-spread in this movement, that assurance is given of its ultimate success. We need not point out, in this connection, the advantages or even the necessity for such a library. The late meeting for its establishment clearly demonstrates that they are acknowledged by all.

We learn that it is contemplated to join, as auxiliary to the Library Association, a bureau for nurses, in which all thoroughly skilled nurses may be registered, and through which the profession may, at any time, acquire all needed information in regard to their capabilities and whereabouts.

Such an institution may become the centre of great usefulness and convenience to the profession and the public. We shall have occasion to refer to the subject again and to place its objects and its needs before the profession.

